# Mechanistic Evaluation of Enhanced Curcumin Delivery through Human Skin In Vitro from Optimised Nanoemulsion Formulations Fabricated with Different Penetration Enhancers

**DOI:** 10.3390/pharmaceutics11120639

**Published:** 2019-12-01

**Authors:** Shereen A. Yousef, Yousuf H. Mohammed, Sarika Namjoshi, Jeffrey E. Grice, Heather A. E. Benson, Wedad Sakran, Michael S. Roberts

**Affiliations:** 1 Therapeutics Research Centre, University of Queensland Diamantina Institute, University of Queensland, Woolloongabba, QLD 4102, Australia; s.yousef@uq.edu.au (S.A.Y.); y.mohammed@uq.edu.au (Y.H.M.); s.namjoshi@uq.edu.au (S.N.); m.roberts@uq.edu.au (M.S.R.); 2 Faculty of Pharmacy, Helwan University, Helwan 11795, Egypt; wedadsakran@hotmail.com; 3 School of Pharmacy and Biomedical Sciences, Curtin Health Innovation Research Institute, Curtin University, Perth 6845, Australia; H.Benson@curtin.edu.au; 4 School of Pharmacy and Medical Sciences, University of South Australia, Adelaide 5000, Australia; 5 Therapeutics Research Centre, Basil Hetzel Institute for Translational Medical Research, The Queen Elizabeth Hospital, Adelaide 5011, Australia

**Keywords:** skin permeation, permeation enhancers, curcumin, nanoemulsions, cutaneous drug delivery, Quality by Design principles

## Abstract

Curcumin is a natural product with chemopreventive and other properties that are potentially useful in treating skin diseases, including psoriasis and melanoma. However, because of the excellent barrier function of the stratum corneum and the relatively high lipophilicity of curcumin (log *P* 3.6), skin delivery of curcumin is challenging. We used the principles of a Quality by Design (QbD) approach to develop nanoemulsion formulations containing biocompatible components, including Labrasol and Lecithin as surfactants and Transcutol and ethanol as cosurfactants, to enhance the skin delivery of curcumin. The nanoemulsions were characterised by cryo-SEM, Zeta potential, droplet size, pH, electrical conductivity (EC) and viscosity (*η*). Physicochemical long-term stability (6 months) was also investigated. The mean droplet sizes as determined by dynamic light scattering (DLS) were in the lower submicron range (20–50 nm) and the average Zeta potential values were low (range: −0.12 to −2.98 mV). Newtonian flow was suggested for the nanoemulsions investigated, with dynamic viscosity of the nanoemulsion formulations ranging from 5.8 to 31 cP. The droplet size of curcumin loaded formulations remained largely constant over a 6-month storage period. The inclusion of terpenes to further enhance skin permeation was also examined. All nanoemulsions significantly enhanced the permeation of curcumin through heat-separated human epidermal membranes, with the greatest effect being a 28-fold increase in maximum flux (*J*_max_) achieved with a limonene-based nanoemulsion, compared to a 60% ethanol in water control vehicle. The increases in curcumin flux were associated with increased skin diffusivity. In summary, we demonstrated the effectiveness of nanoemulsions for the skin delivery of the lipophilic active compound curcumin, and elucidated the mechanism of permeation enhancement. These formulations show promise as delivery vehicles for curcumin to target psoriasis and skin cancer, and more broadly for other skin delivery applications.

## 1. Introduction

Turmeric has a long history of use in folk and alternative medicine, having been used for the treatment of various skin disorders such as acne, skin rashes and warts in Ayurvedic and Chinese systems of medicine [1]. Curcumin is an active compound derived from turmeric with anti-inflammatory, antioxidant, antiviral, antifungal and antibacterial properties. These attributes, along with its low toxicity, make it a promising candidate for a range of clinical applications [2,3,4,5]. Recently, its potential has been recognised for treatment of significant skin diseases such as psoriasis and cancer [6,7]. The inhibitory effect of curcumin on immune pathways critical to the pathophysiology of psoriasis, such as NF-κB and its downstream inflammatory gene products including Th-1-type cytokines (i.e., tumour necrosis factor TNFα, interferon-c) has been demonstrated in in vitro and in vivo studies [6]. Skin cancer is the most common human cancer, and melanoma the most aggressive and resistant to present therapies [8]. Curcumin has been shown to induce autophagy and apoptosis in melanoma, potentially offering a nontoxic option for combinatorial chemotherapy [8]. Curcumin inhibits NF-κB activity to inhibit the growth of melanoma cells, but not normal melanocytes [9], with evidence of inhibition of chemical-mediated carcinogenesis at both initiation and progression stages in preclinical studies [7]. However, curcumin has poor aqueous solubility and poor oral bioavailability due to rapid first-pass metabolism [10], thus limiting its utility as an oral medication [11]. To overcome these limitations, a number of advanced formulation approaches, including nanocrystal and amorphous solid dispersions, liposomes, micelles, nanoparticles, and phospholipid complexes have been investigated to provide longer circulation, better permeability, and resistance to metabolic processes in order to improve oral bioavailability [12,13].

There is a clear opportunity for a skin-delivered curcumin product that could directly target affected tissues in the treatment of skin conditions. However, curcumin exhibits relatively high lipophilicity (log *P* 3.6) and possesses poor passive skin penetration from simple lipid-based vehicles. A number of approaches have been shown to improve skin penetration of curcumin, including soybean phospholipids-loaded liposomes [14], Tween 20 micelles encapsulated in a chitin shell [15] and curcumin loaded chitin nanogels (CCNGs) [16], all aimed at improving curcumin solubility and dispersion in aqueous vehicles.

Nanoemulsions (NE) are heterogeneous systems composed of oil droplets dispersed in aqueous media and stabilised by surfactant molecules [17]. Moreover, they are kinetically stable without any apparent flocculation or coalescence during long-term storage, due to their nanometer sized droplets [18]. Recently, increasing attention has been focused on NE based drug delivery system due to their ease of formulation using low-energy emulsification methods with biocompatible excipients, and unique properties such as smaller droplet size (<200 nm), increasing solubility and dissolution rate, improving diffusion and mucosal permeability [17,19,20,21,22]. Therefore, they offer the advantage of easier scale-up and manufacture compared to the more complex nanosystems. They have been shown to enhance skin delivery of both hydrophilic and lipophilic compounds and could offer an ideal approach for the targeted delivery of curcumin to the skin.

We adopted a partial Quality by Design (QbD) approach for the design of our NE-based formulations of curcumin to drive the rational choice of formulation components to optimise both the physical and skin delivery characteristics of the product. This focused on the effects of a range of surfactants, cosurfactants and oils, including components with the potential to act as skin penetration enhancers. A full implementation of QbD involves the definition of a quality target product profile (QTPP) and critical quality attributes (CQAs) of a drug product, and the accomplishment of risk assessment to identify critical material attributes (CMAs) and critical process parameters (CPPs), the definition of a design space through design of experiments (DoE), the establishment of a control strategy, and the continual improvement and innovation throughout the product life cycle [23,24]. Here we defined a QTPP and identified a number of CQAs for the NE-based topical product that included good safety profile, low irritancy, good compatibility, adequate curcumin solubility, good chemical and physical stability, a wide NE region in the pseudoternary phase diagram, and optimal droplet size. We chose formulation components that are GRASE (generally regarded as safe and effective) to address the safety and irritancy profile of the NE. We prepared two different NE systems based on either the nonionic surfactant Labrasol (hydrophilic lipophilic balance, HLB 12), or the biocompatible phospholipid surfactant lecithin, as both of these have good safety and irritancy profiles. Transcutol and ethanol were chosen as cosolvents for their excellent solubilising capacity for both hydrophilic and lipophilic compounds, good safety and skin penetration enhancing ability [25]. In addition, two terpenes, limonene and eucalyptol, were included to investigate the potential of adding further barrier perturbation.

The objective of the present study was to develop a series of curcumin-loaded NEs using different surfactants, cosurfactants and oils that would provide good long-term stability and enhance the solubility and cutaneous delivery of curcumin. The results of the experiments, including in vitro human epidermal permeation and stratum corneum solubility, were used to provide a mechanistic evaluation of the effect of the NE-based formulations on cutaneous delivery of curcumin. Recent work has begun to examine the efficacy of curcumin in treating skin disease [26], including the targeting of curcumin to specific cell populations in skin regions where diseases such as psoriasis [27] originate, as well as improved NE formulations for skin delivery of curcumin [28]. Results of our work will provide further mechanistic insights to enable us to design optimised delivery systems for cutaneous curcumin delivery.

## 2. Materials and Methods

### 2.1. Chemicals

Curcumin (98% purity), acetonitrile (HPLC grade), citric acid (AR grade), sodium hydroxide liquid (AR grade), ethanol (HPLC grade), methanol (HPLC grade), phosphate buffer saline sachets pH 7.4, +R-limonene (99% purity), eucalyptol (99% purity), isopropyl myristate (IPM), oleic acid, polyethylene glycol 400 (PEG400) and polyethylene glycol 6000 (PEG 6000) were purchased from Sigma-Aldrich Pty. Ltd., Sydney, Australia. Volpo N20 (polyethylene glycol-20-oleyl ether; HLB 14), Ceteth 10 (polyoxyethylene (10) cetylether; HLB 12.9) and corn oil were kindly supplied by Croda, Sydney, Australia. Soy lecithin (99% pure) was purchased from Aventi Polar Lipids Inc., Alabaster, AL, USA. Labrasol^®^ (caprylocaproyl polyoxyl-8 glycerides; HLB 12), Labrafil^®^ M1944CS (Oleoyl polyoxyl-6 glycerides), Capryol™ 90 (propylene glycol monocaprylate type II) and Transcutol^®^ (HP grade: >99.90% purity diethylene glycol monoethyl ether) were kindly supplied by Gattefossé, Saint-Priest, France.

### 2.2. Pseudoternary Phase Diagram Construction

Two different systems were formulated. In System 1, Labrasol was used as surfactant (S), and Transcutol HP and Ceteth 10 were used as cosurfactants (CoS), in the ratio 1:1:0.086 *w*/*w*. For the purpose of the ternary diagram, other ratios of Labrasol and Transcutol were also investigated, but as 1:1 was found to be optimal with satisfactory NE area in presence of ceteth, this was then maintained for the systems that were further characterised. In System 2, soy phosphatidyl choline was used as S and ethanol as CoS in a ratio 1:1 *w*/*w*. First, the mixtures of S and CoS stated above were prepared. Next, mixtures of oil phase (O) with S/CoS were prepared at varying *w*/*w* ratios within the range from 1:9 to 9:1. 1 g of each O/(S/CoS) mixture was titrated with water step by step. At each step, the water/O/(S/CoS) mixture was agitated by a vortex mixer to mix thoroughly, and the sample was visually checked for turbidity against a light versus dark background. If the sample was a clear solution, it was defined as a NE. The corresponding O/(S/CoS) ratio and the water content were recorded at each step of the titration. The boundary point between the NE and the non-NE was determined and recorded experimentally. The NE system pseudoternary phase diagram was constructed using those boundary points in a ternary plot. The data were plotted using Microcal Origin 7 (Microcal Software, Inc.).

### 2.3. Nanoemulsion Preparation

The NEs were prepared using a spontaneous emulsification method [19]. Surfactant and cosurfactant (S/CoS) were mixed together and heated gently in order to melt phosphatidyl choline or ceteth. Then, oil and (S/CoS) were mixed with the aid of a magnetic stirrer to form homogenous, isotropic mixtures that were slowly titrated with the exact amount of water as determined above with continuous stirring to obtain clear and transparent NEs. Drug-loaded NEs were prepared in a similar manner by initially adding 0.5% curcumin to the oil phase with the aid of sonication and vortex mixing to ensure dissolution. Compositions of NEs and control solutions prepared for testing are outlined in Table 1 and Table 2.

### 2.4. Droplet Size and Droplet Size Distribution

Droplet size (Z-average size) and droplet size distribution (polydispersity index: PDI) of NEs were measured by dynamic light scattering (DLS) using a Malvern Zetasizer Nano ZS90 (Malvern Instruments Ltd., Malvern, UK). Light scattering was monitored at a 90 angle and at 25 ± 0.5 °C. The apparent droplet diameter of the NE internal phase was measured using disposable polystyrene cells immediately after dilution with water. Measurements were carried out in triplicate and data presented as mean ± SD.

### 2.5. Droplet Surface Charge (Zeta Potential)

The droplet surface charge or Zeta potential (ZP) was determined on preparation (1 Day) by laser Doppler electrophoresis using the Zetasizer Nano ZS90 (Malvern Instruments Ltd., Malvern, UK) at 25 °C. In this case all formulations were prepared using an aqueous phase containing sodium chloride (0.001 M) in order to ensure constant conductivity. The formulations were stored at 25 °C and reanalysed after 6 months. Measurements were performed in triplicate and data presented as mean ± SD.

### 2.6. pH Measurement

The pH of both blank and drug-loaded NEs was measured with a Meridian pH meter (Denver Instrument, Bohemia, NY, USA) at 25 °C. Measurements were carried out in triplicate and data are expressed as mean ± SD.

### 2.7. Refractive Index

The refractive index values of all NEs were measured using an ATAGO Model PAL-RI digital refractometer (ATAGO, Tokyo, Japan). Measurements were conducted in triplicate at room temperature by adding 0.3 mL of each NE into the testing well. Data are presented as mean ± SD.

### 2.8. Rheological Properties

The rheological properties of the NEs were determined with a DHR-3 Rheometer equipped with TRIOS data processing software (TA Instruments, New Castle, DE, USA). Flow curves were recorded for all NE systems. The flow properties of both blank and drug-loaded NEs were investigated by measuring the dynamic viscosity *η* (in Pa·s) under shear stress. Rheological experiments were performed in a controlled-rate mode. A controlled shear rate *γ* was employed at a constant temperature of 25 ± 0.5 °C to determine the viscosity of the samples as a function of the shear rate ranging from 5 s^−1^ to 125 s^−1^. The rheological behaviour of each system was evaluated by plotting the shear stress versus the shear rate values obtained. The systems which showed proportionality in both parameters (*R*^2^ values ≥ 0.99) were considered to be Newtonian fluids. All measurements were performed in triplicate (*n* = 3). Data are presented as mean ± SD.

### 2.9. Electrical Conductivity (EC)

The NE microstructure was assessed by EC tests. EC values of each sample were measured using a digital electrical conductivity meter (Digitor Q1563, DSE, Brisbane, Australia) with AgCl and Ag conductivity electrodes at room temperature. The oil/surfactant mixture (3 g) with O/(S/CoS) ratio 1/9 was titrated by the aqueous phase (0.9% NaCl solution) step by step. At each step, 1 mL of the sample was used for EC measurement at room temperature. Measurements were performed in triplicate and data are presented as mean ± SD.

### 2.10. Cryo-Scanning Electron Microscopy (Cryo-SEM)

NEs were loaded into copper rivets and plunge frozen in liquid propane at a temperature of −180 °C with a Reichert KF80 cryofixation system (Leica, Wetzlar, Germany). Samples were stored in liquid nitrogen before being transferred into the cryostage (Alto 2500, Gatan, Inc., Abingdon, UK) of the microscope (JSM-6700F, JEOL Ltd., Tokyo, Japan). After fracturing the sample with a knife, it was viewed at −140 °C and an accelerating voltage of 2.5 kV.

### 2.11. Stability of Curcumin in the Receptor Medium

Standard solutions of curcumin (5, 10, 12.5 and 25 µg/mL) were prepared in a 6% Volpo aqueous solution and stored at 35 ± 0.5 °C for 24 h. Samples removed at 1, 6, 12 and 24 h were assayed for drug content by HPLC.

### 2.12. Physical and Chemical Stability of Curcumin and Nanoemulsion Formulations

The physical stability of NEs was estimated by determination of droplet size over 6 months. All formulations were stored in a tightly closed container protected from light by aluminium foil at 25 °C, and visually inspected against a dark/light background every 4 weeks for signs of instability. Chemical stability of the drug loaded NEs was evaluated by measuring the amount of curcumin in the loaded formulations immediately and after storage at 25 °C for 6 months. Curcumin content was determined using HPLC by dissolving 10 mg of the NEs in 1 mL of methanol followed by vortexing for 20 s. The ZP and pH values of loaded NEs were also measured after 6 months of storage and compared to those measured at day 1. All measurements were in triplicate for three different samples.

### 2.13. HPLC Quantification of Curcumin

A reversed phase HPLC method was developed using a SIL-20A autosampler, CBM-20A system controller, SPD-20A UV/vis detector and LC-20AD pump (Shimadzu Corp., Kyoto, Japan). Chromatographic separation was performed on a Luna C18(2) column (150 × 4.6 mm, 5 μm: Phenomenex Inc., Torrance, CA, USA) and sample injection volume of 20 μL. All samples were analysed under isocratic elution at a flow rate of 1.0 mL/min, the detection wavelength was set at 430 nm and the retention time of curcumin was 3 min.

The freshly prepared mobile phase of 75:25 acetonitrile: citrate buffer (pH = 3) was degassed for 15 min before analysis. Stock solutions of curcumin (1 mg/mL) was prepared in methanol. The curcumin stock solution was diluted with methanol to working solutions ranging from 0.0097 to 100 μg/mL. All solutions were protected from light and stored at −20 °C between experiments. Quality control (QC) samples were independently prepared at three level concentrations of 1, 40 and 100 µg /mL. The QC samples were stored at −20 °C and brought to room temperature before use. The lower limit of quantitation (LLOQ) of the assay was 0.0097 µg/mL. The reproducibility of LLOQ was determined by examining triplicate LLOQ samples independent of the standard curve, and the accuracy and precision of LLOQ and QCs were within 10%. Calibration curves of three different lots of curcumin were linear in the range of 0.0097–100 µg/mL with (*R*^2^ = 0.999).

### 2.14. Human Skin Preparation

Skin samples were obtained with informed consent from female patients undergoing elective abdominoplasty, and approval from the University of Queensland Human Research Ethics Committee (HREC/16/QPAH/064 and 2008001342, approved 27 August, 2017). The procedures were conducted in compliance with guidelines of the National Health and Medical Research Council of Australia. Full thickness skin was prepared by removal of subcutaneous fat by blunt dissection. Heat separation was used to separate epidermal membranes by immersing full thickness skin in water at 60 °C for 1 min, to allow the epidermis to be teased away from the dermis [29]. Stratum corneum was prepared from the epidermal membranes by trypsin digestion [30]. The epidermis was floated overnight in a solution of 0.01% trypsin in phosphate buffer saline at 37 °C. The digested viable epidermis was gently scraped off with cotton buds and the remaining stratum corneum was rinsed several times with distilled water. The isolated stratum corneum membranes were dried with absorbent paper and placed flat between parafilm^®^ sheets covered with aluminum foil. All skin membranes were stored frozen at −20 °C until use, mostly within one week.

### 2.15. Determination of the Solubility of Curcumin in NE Formulations (S_V_)

The solubility of curcumin in each formulation (*S_V_*) was determined by adding excess curcumin in 5 mL of each NE or control solution until an excess amount remained. The samples were incubated in a water bath at 32 °C for 48 h with continuous agitation, then centrifuged at 16,160× *g* for 10 min at 32 °C. Supernatant solutions were filtered through a 0.45 μm membrane filter (Acrodisc^®^ syringe filter with nylon membrane: Gelman Sciences Pty Ltd., Cheltenham East, Australia) and diluted with methanol to quantify the amount of curcumin by HPLC.

### 2.16. Determination of Solubility of Curcumin in the Stratum Corneum (S_SC_)

To determine the solubility of curcumin in the stratum corneum from the various vehicles, preweighed discs of stratum corneum (four replicates) were incubated in 1 mL saturated formulation at 32 °C for 24 h [30,31]. The stratum corneum discs were then removed and blotted dry before being further incubated with 1 mL of methanol for 24 h at 32 °C to enable complete extraction of curcumin. *S*_SC_ was determined from the amount of curcumin recovered in the extraction fluid measured by HPLC divided by the thickness and area of the stratum corneum [31].

### 2.17. In Vitro Skin Permeation and Distribution Study

In vitro skin permeation studies were performed with epidermal membranes in Franz-type vertical diffusion cells with an effective diffusion area of 1.33 cm^2^ and approximately 3.4 mL receptor chamber capacity. The epidermal membrane was cut into discs and mounted between the donor and receptor compartments with the stratum corneum side facing the donor chamber. The donor and receptor compartments were filled with 0.1 M PBS (pH 7.4) and the integrity of the epidermal membrane was assessed by measuring the resistance between the donor and the receptor compartment with Ag/AgCl electrodes attached to a multimeter (Digitor Q1563, DSE, Brisbane, Australia). The epidermis was discarded if the resistance was lower than 20 kΩ·cm^2^. After resistance measurements, the PBS was removed from both compartments and the receptor compartment was filled with Volpo 6% in water and immersed in a water bath at 35 ± 0.5 °C to maintain the skin surface temperature at 32 ± 0.5 °C. The donor solution consisted of 1 mL of the NE containing 0.5% *w*/*w* curcumin or control formulations saturated with curcumin. The donor compartment was covered with Parafilm^®^ to prevent evaporation. At predetermined time points (1, 2, 3, 4, 5, 6, 8 and 24 h), 200 μL of the receptor phase was withdrawn and replaced with an equal volume of fresh Volpo 6% receptor medium. The curcumin content in all samples was determined by HPLC.

### 2.18. Data Analysis

The cumulative amount (*Q*, μg/cm^2^) of curcumin penetrating through an area of 1.33 cm^2^ was plotted against time (*t*). Steady state flux *J*_SS_ (μg/cm^2^/h) was determined from the slope of the linear portion of the cumulative amount (*Q*) versus time (*t*) plot.

*J*_SS_ = *k*_p_ × *C*_v_ (1)

The maximum flux (*J*_max_) that would be applicable to saturated solutions can be estimated from the experimental steady state flux corrected for the known solubility in the formulation by Equation (2) [31].

*J*_max_ = *J*_SS_ × *S*_v_ / *C*_v_ (2)

where *S*_V_ is the solubility in the formulation and *C*_V_ is the experimental concentration used.

The apparent diffusivity of solute in the skin divided by path length (D*) was calculated from the maximum flux and the solubility of the active compound in the SC according to Equation (3) [31].

D* = *J*_max_*/ S*_SC_ (3)

### 2.19. Statistical Analysis

All experiments were analysed by one-way analysis of variance (ANOVA) with post-hoc comparisons (Tukey) using GraphPad Prism 6 (GraphPad Software Inc., La Jolla, CA, USA); a result was considered significant when *p* < 0.05. Comparisons were made between the NE formulations and controls, as well as between the different NE formulations for all the permeation parameters (*Q*_24_, *J*_max_, D* and *S*_SC_) and for other experimental parameters related to physicochemical characterisation of the NEs.

## 3. Results

### 3.1. Pseudoternary Phase Diagram

**Figure 1:**
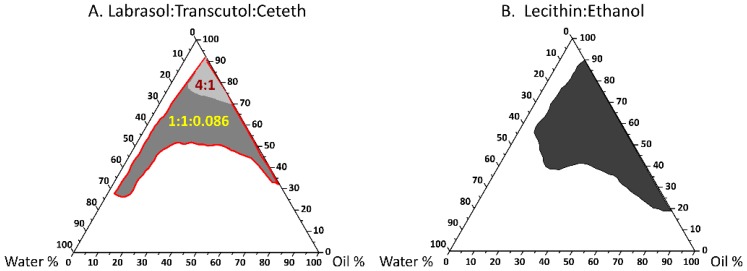
Ternary phase diagram of the oil, surfactant/cosurfactant mixture and water. The black, light and dark grey parts represent the nanoemulsion areas. (**A**) Labrasol/Transcutol system at a 4:1 ratio, represented by light grey area. Addition of Ceteth at a ratio of 1:1:0.086 (Labrasol/Transcutol/Ceteth) increased the nanoemulsion area to include the dark grey region (the total nanoemulsion area represented by red borders); (**B**) Lecithin/ethanol system, at a ratio of 1:1.

The shaded area refers to the transparent NE region in the constructed pseudoternary phase diagrams (Figure 1A,B). The combination of Labrasol with Transcutol as cosurfactant resulted in a very small NE area (i.e., poor emulsification, not shown). A 4:1 Labrasol:Transcutol ratio provided improved emulsification with a slightly larger shaded area than the 1:1 ratio, so this optimal ratio was plotted in Figure 1A as the pale grey region. Addition of Ceteth 10 (ratio 1:1:0.086) markedly increased the NE area, with the total NE area shown by the red boundary (Figure 1A). At the high surfactant content area, a large amount of water was able to be solubilised without causing phase separation. For example, with the Labrasol/Transcutol/Ceteth system, the oil–surfactant mixture [O/(S/CoS)] ratio of 1/9 can be diluted to higher than 70% (*w*/*w*) water content and remain as a nanoemulsion. Along this water dilution line, it is possible to study the complete course of nanoemulsion microstructural changes. For the lecithin:ethanol system (Figure 1B), a satisfactory NE area was achieved at a ratio of 1:1 and this system could be diluted up to approximately 50% (*w*/*w*) water.

### 3.2. Nanoemulsion Characterisation

#### 3.2.1. Droplet Size, Size Distribution (PDI) and Zeta Potential (ZP)

**Figure 2:**
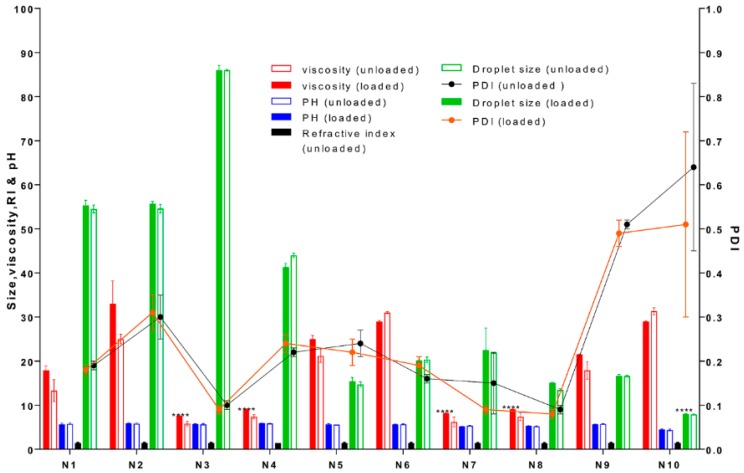
Physical characterisation of NEs (N1–N10) with and without curcumin (loaded and unloaded). No significant differences (*p* > 0.05) in viscosity (cps), pH and average droplet size (Z-average, nm) were seen for loaded formulations compared to unloaded (*n* = 3, mean ± SD). Significant differences (**** *p* < 0.0001) in viscosity were observed for formulation with terpenes (N3–4, N7–8) compared to other oils. A significant difference (**** *p* < 0.0001) in droplet size for formulation N10 compared to other formulations and a high poly dispersity index (PDI) for N10, indicating a wide range of size distribution. Refractive index (RI) was measured for unloaded formulations only.

**Figure 3:**
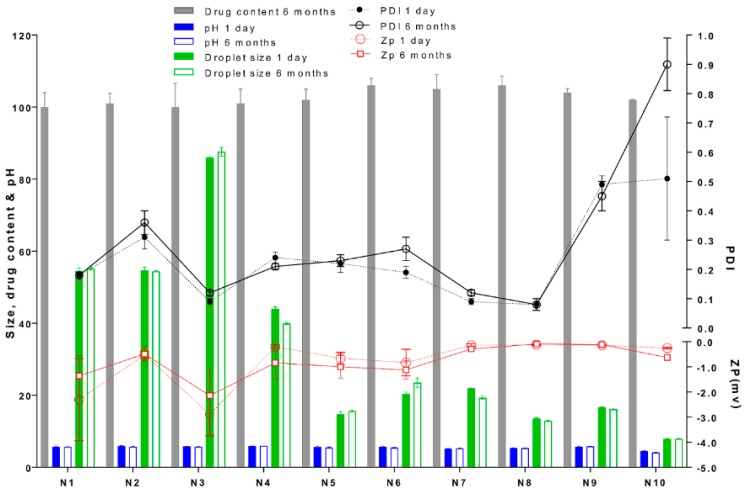
Physical and chemical characterisation of curcumin-loaded nanoemulsions after long term storage for 6 months at 25 °C compared to 1-day. No significant difference (*p* > 0.05) in zeta potential (ZP, mV), drug content (%*w*/*w*) droplet size (nm), pH, was observed after storage compared to 1-day preparation, with exception of formulation N10 showed higher polydispersity index (PDI), indicating aggregation.

All formulations were highly fluid and homogeneous upon visual inspection. Lecithin-based NEs were significantly smaller (**** *p* < 0.0001) than Labrasol-based NEs for all the different oil tested (≅20 and 50 nm respectively: Figure 2). The PDI ranged from 0.09 to 0.64 in the unloaded NEs, as seen in Figure 2. The ZP values for all NEs at preparation on day one were low (range: −0.12 to −2.98 mV: Figure 3) and not affected by the surfactant and cosurfactants used. The incorporation of curcumin did not significantly affect the droplet size, size distribution or surface charge of the NEs (*p* > 0.05).

#### 3.2.2. pH and Refractive Index (RI) of NE Formulations

The NEs had a pH range of 4.5 to 5.9, and the formulation with oleic acid N10 showed the lowest pH value. The RI of unloaded NEs was in the range of 1.41 to 1.44 (Figure 2).

#### 3.2.3. Rheological Properties

All NE formulations exhibited Newtonian flow behaviour, as the viscosity remained constant at different shear rates. Dynamic viscosity of the NE formulations ranged from 5.8 to 31 cPs, with no significant increase (*p* > 0.05) in viscosity on curcumin addition, as seen in Figure 2. Formulations with terpenes showed significantly lower viscosities (**** *p* < 0.0001) compared to the Labrafil and oleic acid systems.

#### 3.2.4. Electrical Conductivity

**Figure 4:**
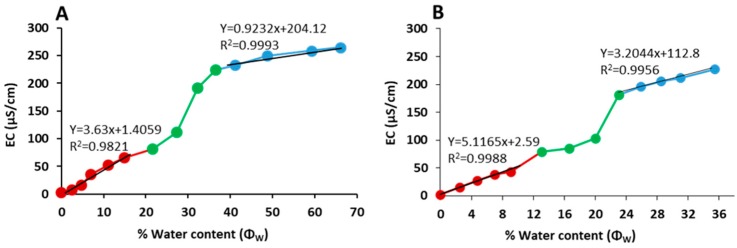
The plot of nanoemulsion electrical conductivity (EC) versus aqueous content; (**A**) Electric conductivity (EC) Labrasol/Transcutol system; (**B**) Electric conductivity (EC) For lecithin /ethanol system; red circles for low water content, green and blue for median and high water content, respectively.

All NEs were poor electrical conductors, with conductivity ranges of 0.48 to 0.55 µS/cm and 1.32 to 1.38 µS/cm for the Labrasol and lecithin systems, respectively. The oil–surfactant mixture O/(S/CoS) ratio of 1:9 was diluted gradually by the 0.9% (*w*/*v*) NaCl aqueous phase, and the electrical conductivity (EC) values measured following each dilution plotted vs. percent water content (*Φw*), as shown in Figure 4A,B. The EC vs. *Φw* curve can be divided into three parts (low, medium and high aqueous content) and used to assess the system microstructure, as previously described [32]. At low aqueous content, the microstructure is perceivably w/o, resulting in a low conductivity region in which the EC vs. *Φw* curve appears linear. As the aqueous content *Φw* increases, the w/o droplets increase in number and start to aggregate, resulting in a drastic increase of EC. Therefore, the turning point from linear to non-linear increase on the EC vs. *Φw* curve corresponds to the transformation from w/o droplets to a bicontinuous NE [32]. At high aqueous content, the microstructure is o/w where the conductive entity is the continuous aqueous phase and the EC reverts to a linear increase again.

The EC vs. *Φw* curves for our systems approximated linear fitting with good correlation coefficients at low and high *Φw* (Figure 4A,B). The curve at the median *Φw* region is nonlinear, corresponding to a bi-continuous NE microstructure. Based on the EC vs. *Φw* curves the transformations from w/o to bi-continuous and from bi-continuous to o/w occur at water content about 22% and 40%, respectively, for the Labrasol system (Figure 4A), and about 16% and 26%, respectively, for the lecithin system (Figure 4B).

#### 3.2.5. Microscopic Evaluation Using Cryo-SEM

**Figure 5:**
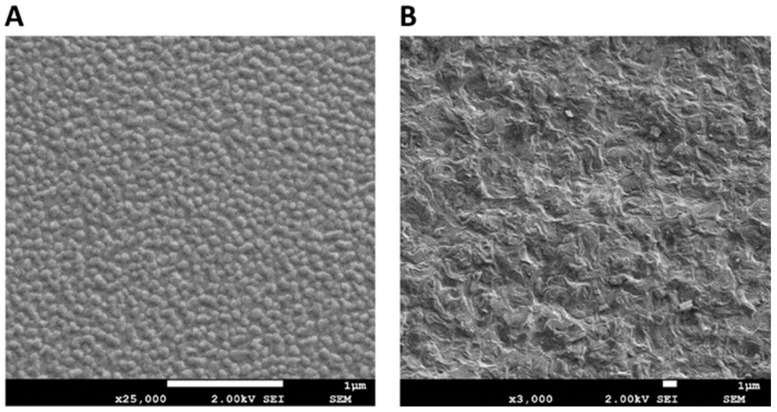
(**A**) Cryo-SEM micrographs of formulation N3 Labrasol/Transcutol system; (**B**) Cryo-SEM micrographs of formulation N7 lecithin/ethanol system. Image magnification and scale bars representing 1 μm are shown on each figure.

Typical cryo-SEM images of NE formulations N3 and N7 are shown in Figure 5A,B, respectively. Figure 5A shows a typical electron micrograph of a w/o system (N3), supporting the microstructural prediction of the conductivity measurements for the droplet nanoemulsion samples of the Labrasol/Transcutol systems. In contrast, the cryo-SEM of the lecithin-based system N7 (Figure 5B) shows a spongy structure typical of a bicontinuous nanostructure.

### 3.3. Stability Studies

#### 3.3.1. Stability of Curcumin in the Receptor Medium

The amount of curcumin remaining in 6% Volpo in water at 0, 1, 6, 12 and 24 h was >99%, indicating that 6% Volpo is a suitable receptor medium for curcumin in the skin studies.

#### 3.3.2. Physical and Chemical Stability of Curcumin and the NE Formulations

The droplet size and size distribution of curcumin-loaded Labrasol and lecithin-based NE formulations remained largely constant over 6 months (Figure 3). Curcumin content was 100 to 106 percent of the initial amount for all NEs (Figure 3) and no degradation products were detected by HPLC demonstrating that curcumin was stable within all NEs systems over 6 months. There were no significant changes (*p* > 0.05) in pH or ZP values after 6 months of storage.

### 3.4. In Vitro Permeation of Curcumin across Human Epidermal Membranes

**Figure 6:**
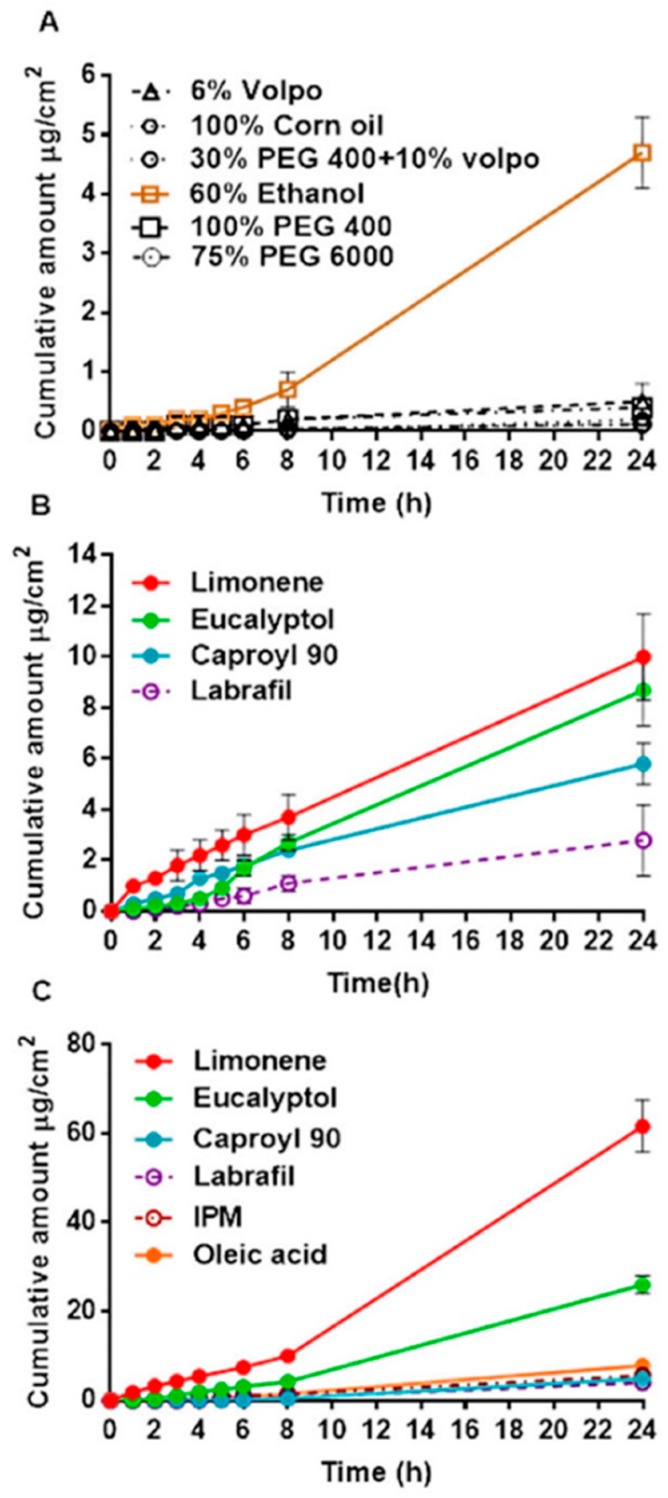
In vitro percutaneous permeation through epidermal human skin: (**A**) Controls & 60% ethanol; (**B**) Labrasol system; (**C**) Lecithin system.

The cumulative amount of curcumin penetrated across human epidermal membrane (μg/cm²) was plotted as a function of time (h) and the slope yielded the pseudo steady-state flux *J*_SS_ (μg/cm^2^/h). The cumulative amount of curcumin penetrated vs. time for control vehicles, Labrasol-based NEs and lecithin-based NEs is shown in Figure 6A–C, respectively. The 60% aqueous ethanol solution showed significantly higher cumulative curcumin penetration at 24 h than the other control solutions, as seen in Figure 6A. The cumulative amount of curcumin penetrated over 24 h was significantly greater from all NE systems compared to PEG, 6% Volpo and corn oil control vehicles, with increases as much as >100-fold (Figure 6B,C). It is also apparent that the incorporation of terpenes further enhances curcumin penetration compared to other oil phases. In the case of the lecithin-based NE formulations, this enhancement was statistically significant (** *p* < 0.05), but for the Labrasol-based NE formulations containing terpenes; the enhancement was significantly different compared to Labrafil (* *p* < 0.05). The greatest enhancement was seen with limonene for both the Labrasol and lecithin-based NE systems (Figure 6B,C).

### 3.5. Effect of Formulation on Curcumin Solubility, Solubility in Stratum Corneum, Maximum Flux, Permeability Coefficient and Derived Diffusivity

Table 3 shows the estimated curcumin saturated solubility in the formulations (*S*_V_), solubility in the stratum corneum (*S*_SC_), partition coefficient (*K*_SC_), steady state flux (*J*_SS_) from the cumulative amount penetrated vs. time curve, permeability coefficient *k_p_* (cm/h) calculated from Equation (1), maximum flux (*J*_max_) calculated from steady state flux using Equation (2), and diffusivity per path length (D*) derived from the *J*_max_ and *S*_SC_ using Equation (3).

Curcumin solubility was highest in the Labrasol-based NEs (26.7–58.3 mg/mL), followed by the lecithin-based NEs and 100% PEG400 vehicle (10.9–18.6 mg/mL), compared to the other control vehicles (0.7–3.7 mg/mL). Curcumin *J*_SS_ was significantly greater for the aqueous ethanol solution (Ce) and NE formulations containing enhancers compared to PEG, 6% Volpo/water and corn oil control solutions (**** *p* < 0.0001).

Curcumin *J*_max_ from NEs was significantly greater than for all control solutions (**** *p* < 0.0001), and greatest from lecithin-based NEs containing terpenes, as seen in Table 3. Enhancement of curcumin penetration was 2.5 to 27.6-fold greater for NEs than 60% ethanol (Ce), and >100-fold compared to other control vehicles. Curcumin solubility in the SC was similarly increased for all NE formulations containing enhancers, and significantly higher (**** *p* < 0.0001) than all control vehicles.

**Figure 7:**
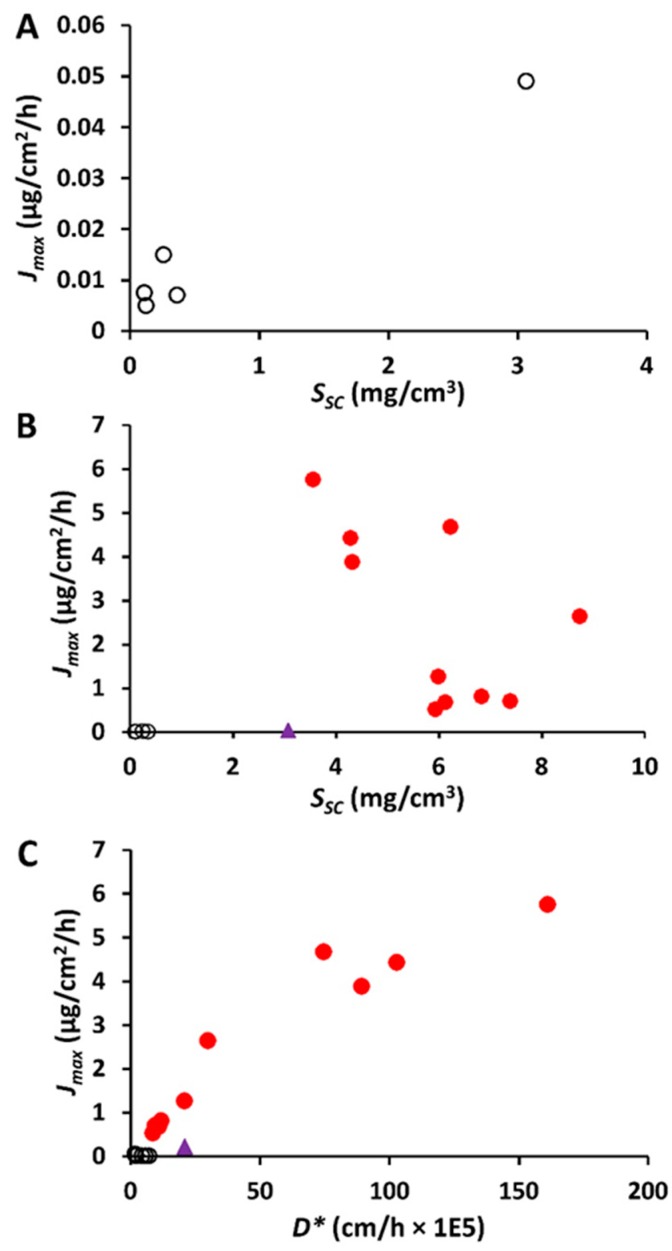
Impact of stratum corneum (SC) solubility and apparent diffusivity D* on the maximum flux *J*_max_ for curcumin. (**A**). *J*_max_ versus *S*_sc_ for control vehicles only excluding 60% ethanol; (**B**). *J*_max_ versus S_sc_; (**C**). *J*_max_ versus D*. Black symbols—for control vehicles; purple symbols—60% EtOH/water; red symbols—nanoemulsion formulations. It is evident from Figure 7C that the *J*_max_ is better related to D*.

To investigate the determinants of increased flux and provide an insight into the mechanism of action of these formulations, we examined the relationships between some of the parameters in Table 3. Rearranging Equation (3), we obtain *J*_max_ = *S*_SC_ × D*, indicating that if D* is constant, there should be a linear relationship between *J*_max_ and *S*_SC_ (with a slope of D*, or *D*/*h*). With the exception of 60% ethanol, there was a minimal variation in D* between the control vehicles (Table 3). Figure 7A illustrates the observed relationship between *J*_max_ and *S*_SC_ for curcumin and the control vehicles, with *R*^2^ value 0.88. The 60% ethanol vehicle and NEs with penetration enhancers show elevated *J*_max_ values that do not fit the relationship seen with the control vehicles, reflecting the effects of these formulations on diffusivity. The mechanism of curcumin penetration enhancement for the NEs with enhancers is more clearly demonstrated in Figure 7B,C. In Figure 7C, it is evident that the *J*_max_ for curcumin is better related to its altered diffusivity D* associated with the various formulations *R*^2^ = 0.89. In contrast, the *R*^2^ of *J*_max_ versus *S*_SC_ of the formulations is 0.0963 (Figure 7B).

## 4. Discussion

Nanoemulsions offer a promising skin delivery system for both hydrophilic and lipophilic compounds [33]. Many properties related to their composition and physicochemical characteristics can influence NE suitability and efficacy, and increasing our understanding of these properties will extend the application of NE-based skin products for a range of dermatological and cosmetic purposes. In our study, NEs were developed using the principles of HLB and pseudoternary phase diagrams, and incorporating a range of components including known chemical penetration enhancers, have been physically and chemically characterised, and their human skin penetration determined. We have shown that well-formulated NE systems can effectively deliver the lipophilic natural compound curcumin into the skin, and that the incorporation of penetration-enhancing chemicals within the systems can enhance skin delivery. We have also elucidated the mechanism of penetration enhancement of these systems, which should aid in the rational design of NEs for skin delivery.

As the type of NE formed and its CQA depend on the properties of the surfactant and cosurfactant [34,35], we investigated two different NE systems based on the nonionic surfactant Labrasol (HLB 12) and lecithin, a phospholipid that acts as a biocompatible surfactant with an HLB value of 8. In System 1, we coupled Labrasol with Transcutol, a highly purified form of diethylene glycol monoethyl ether, as the cosurfactant chosen for its excellent solubilising capacity for both hydrophilic and lipophilic compounds, good safety and skin penetration enhancer ability [25]. System 2 combined lecithin with ethanol, again to enhance solubility and skin permeation [36]. Phospholipid vehicles are reported to enter the stratum corneum lipid bilayers, perturbing their structure and increasing skin hydration, consequently increasing drug permeation [37]. Ethanol is a well-established skin penetration enhancer capable of increasing solubility and decreasing stratum corneum barrier properties [38,39]. In addition, two terpenes, limonene and eucalyptol, were included to add further barrier perturbation.

The two systems had similar surfactant and oil/water content, and a range of oil phase ingredients were used, including the terpene-based penetration enhancers limonene and eucalyptol. Thus, the design of these systems allowed us to investigate the effect of excipients with known solubility and skin penetration enhancement ability, incorporated in various combinations within NE systems capable of presenting both the curcumin active and formulation components to the stratum corneum surface in a highly efficient small droplet format.

We examined the effect of these formulations on attributes including curcumin solubility, chemical and physical stability, NE region in the pseudoternary phase diagram, and optimal droplet size. All systems were w/o NEs, as demonstrated by the refractive index being close to that of the oil phase ≈ 1.44 (Figure 2) and the effect of dilution with sodium chloride solution on electrical conductivity, where distinct regions existed corresponding to w/o and o/w microstructures and their microstructural transition points (Figure 4A,B). All systems generated NEs with adequate to good regions in the pseudoternary phase diagram and droplet sizes in the ≈ 20–50 nm range, showing their suitability as skin products. In all cases, nonionic surfactants were chosen to minimise irritation, although there is the risk that as these systems do not have high zeta potentials, their droplets do not benefit from charge-related repulsion and there is greater risk of aggregation. However, microstructure visualisation of some optimised formulations by cryo-SEM showed globular structures without aggregation, and confirmed the sizing measurements from DLS and PDI. Only the NEs containing oleic acid and isopropyl myristate (IPM) showed a high polydispersity value, suggesting that with these exceptions, the formulations were less likely to be unstable over time due to droplet aggregation. This was confirmed by good physical and chemical stability over 6 months of storage (Figure 3). The results of our rheological studies showed that the NEs followed Newtonian behaviour, which supports the existence of small droplets in liquid systems. Non-Newtonian flow may introduce some unpredictability in skin permeation, as demonstrated by Cross et al. [40], where finite dose application of thicker formulation enhanced the penetration of the sunscreen Oxybenzone. In this work, where we were assessing the effects of nanosystem formulation parameters on skin permeation, it was preferable to eliminate additional complications due to non-Newtonian flow behaviour. Curcumin was easily incorporated into all NE formulations and did not alter their physical properties, demonstrating its good solubility in the formulation components. Overall, both NE systems provided enhanced curcumin solubility compared to the range of control vehicles and provided small droplet size to aid presentation to the skin surface and stable products.

Skin penetration of curcumin was enhanced for all NE-based formulations compared to control vehicles. As expected, among the control vehicles, curcumin skin penetration was highest with 60% aqueous ethanol, which exhibited a flux value approximately 20 times greater than any of the other control vehicles, as seen in Table 3. Ethanol at this percentage has been shown to extract skin lipids and evaporation of the vehicle could also lead to curcumin super saturation at the skin surface, as we noted a high retention of curcumin in the stratum corneum and viable epidermis from this vehicle (data not shown). The ethanol control was therefore used as our control comparator for determining penetration Enhancement Ratio (ER) values with the NE-based formulations. When comparing the two NE systems, without the incorporation of terpenes, there was minimal difference in skin penetration. Addition of terpenes resulted in a marked increase in curcumin skin penetration for both NE systems, as seen in Table 3, providing ER values in the range 11.0–27.6.

We next considered the mechanism of skin penetration enhancement and present evidence of three mechanisms which may be acting to varying degrees. First, enhanced solubility of curcumin in the applied vehicle, as seen in Table 3, increasing the effective dose applied to the skin surface. Second, increase in surface area coverage due to distribution of the curcumin in the nano-sized NE droplets that aids transfer of the lipophilic curcumin from the formulation to the stratum corneum [41]. Third, modification of the solvent nature of the stratum corneum caused by fluidisation of the stratum corneum lipids, improving curcumin solubility and partitioning within the stratum corneum [37,42]. Terpenes and fatty acids are known to disrupt the stratum corneum barrier by multiple mechanisms, including dissolution of stratum corneum lipids [42,43]. These multiple mechanisms can lead to enhanced permeation of a solute, such as curcumin, even if the *S*_v_ in a particular vehicle is lower than in others (for example, N7 & N8) [44,45]. Table 3 shows that N7 and N8 have the highest and third-highest ERs, respectively, and the highest curcumin diffusivities, with only moderate *S*_v_ values. In summary, the enhanced skin delivery with nanoemulsion formulations compared to controls is due to a combination of mechanisms, including increased curcumin solubility in the vehicle, nano-sized droplets ensuring efficient presentation to the stratum corneum surface, enhanced thermodynamic activity and direct enhancer effects on the stratum corneum barrier.

While it is clear that inclusion of a terpene enhanced the skin delivery of curcumin from the NE formulations, there was no clear difference between the terpenes examined in our study, as seen in Table 3. There have been many differing views of the relative effectiveness of terpenes over a wide range of different solutes [46,47,48]. For example, hydrocarbon terpenes, especially limonene, were reported to be as effective as Azone in increasing the flux of indomethacin (log *P* 4.27) across rat skin, while cyclic ether terpenes, such as eucalyptol, were ineffective [49]. Higher curcumin flux (*J*_ss_) at the concentration used (0.5% *w*/*w*) was noted with limonene regardless of the type of S/CoS used. However, our study also demonstrated that cyclic ether terpenes (eucalyptol) promoted curcumin permeation across human skin consistent with that reported for the lipophilic compound tamoxifen [50].

To examine the NE-induced skin permeation enhancement of curcumin, we estimated the solute maximum (or saturated) fluxes (*J*_max_), since *J*_max_ is independent of the vehicle and dependent solely on the thermodynamic activity of the solute in the vehicle, provided the vehicle or solute does not alter the properties of the membrane [31,51]. This concept was initially highlighted by the work of Twist and Zatz [52], who showed the same flux for methyl paraben across synthetic membranes, regardless of its solubility in a range of different vehicles. Our results for the control solutions are consistent with these findings, where low *J*_max_ for curcumin was seen for each control solution not affecting skin properties across a range of vehicle solubilities. In contrast, the *J*_max_ values for NEs, and to some extent, the 60% aqueous ethanol control solution, were much higher than the other control vehicles, as seen in Table 3, suggesting enhanced skin permeation as well as alteration of the stratum corneum properties.

To investigate the mechanism of penetration enhancement of these systems, we estimated the underlying *J*_max_ determinants, i.e., stratum corneum solubility (*S*_SC_*)* and stratum corneum diffusivity (D*). Ethanol, when used as a cosurfactant, has been shown to extract stratum corneum lipids and perturb barrier function, improving the permeation of many lipophilic drugs such as curcumin through skin by partitioning between the vehicle and stratum corneum [53,54]. Ethanol has also been reported to act synergistically by increasing the partitioning of terpenes into skin to enhance penetration of both hydrophilic and lipophilic drugs [55,56].

Curcumin *S*_SC_ was enhanced by NEs, but there was no clear relationship between *J*_max_ and *S*_SC_ (Figure 7B). In contrast, there was an apparent linear relationship between effective diffusivity D* in the epidermal membrane and *J*_max_ for curcumin (Figure 7C), suggesting that the NE formulations modified the stratum corneum lipids and that this increased D* was, in turn, associated with an enhanced flux. In general, diffusivity will be independent of solute lipophilicity for vehicles that do not affect the stratum corneum [57,58]. However, when a vehicle does affect the stratum corneum, there may be varying impact for different types of solutes. We showed that curcumin diffusivity-mediated flux is much more pronounced for terpenes and Caproyl 90 compared to fatty acids, IPM and oleic acid, as seen in Table 3, despite the latter vehicle components also having well-established skin penetration enhancement properties. This is in agreement with previous reports from our laboratory and others [59,60], including reduced diffusivity for the more lipophilic phenols (above log *P* of 3.0) from IPM [59], and a moderate diffusion-enhancing effect of oleic acid compared to d-limonene for solutes with a range of lipophilicities [60]. In the latter study, oleic acid increased the partitioning of butyl paraben (log *P* 3.6) compared to limonene, similar to our findings for curcumin.

## 5. Conclusions

NEs prepared by the spontaneous emulsification method improved curcumin solubility and skin penetration. The inclusion of limonene in the NEs N3 and N7 and eucalyptol in N4 and N8 enhanced curcumin skin penetration by 11, 27, 22 and 21-fold respectively, compared to the 60% ethanol/water positive control, as seen in Table 3. Mechanistic investigation suggests that the enhanced transdermal curcumin delivery was due to a reduction in the diffusion barrier of the stratum corneum. This resulted from NE-based increase in both the stratum corneum solubility and stratum corneum diffusivity of curcumin. The enhanced solubility resulted from the solute being carried into the stratum corneum with the formulation. This study provides clear evidence of the efficacy of terpene containing NE formulations for skin delivery of lipophilic compounds and the mechanism of their action. Our studies using human skin in vitro revealed a potential for targeted therapeutic delivery of curcumin from the optimised formulations to deeper skin layers.

## Figures and Tables

**Table 1:** Control vehicles (C1 to C5 & Ce, % *w*/*w*). All are saturated solutions of curcumin except for PEG 400 neat liquid, in which curcumin solubility exceeds 0.5% *w*/*w*, see Table 3.

Formulation	PEG 6000	PEG 400	Ethanol	Volpo	Water	Corn Oil
C1	75	------	------	------	25	------
C2	------	100	------	------	------	------
C3	------	30	------	10	60	------
C4	------	------	------	6	94	------
C5	------	------	------	------	------	100
Ce	------	------	60	------	40	------

**Table 2:** Formulation content: Nanoemulsions (NEs) N1–N10, curcumin concentration was 0.5% *w*/*w* (5 mg/mL).

Formulation	Lecithin	Ethanol	Labrasol	Transcutol HP	Ceteth 10	Oil Phase %	Water
N1	------	------	35	35	3	17 (Caproyl 90)	10
N2	------	------	35	35	3	17 (Labrafil)	10
N3	------	------	35	35	3	17 (Limonene)	10
N4	------	------	35	35	3	17 (Eucalyptol)	10
N5	35	35	------	------	------	15 (Caproyl 90)	15
N6	35	35	------	------	------	15 (Labrafil)	15
N7	35	35	------	------	------	15 (Limonene)	15
N8	35	35	------	------	------	15 (Eucalyptol)	15
N9	35	35	------	------	------	15 (IPM)	15
N10	35	35	------	------	------	15 (Oleic acid)	15

**Table 3:** Solubilities and skin permeation parameters of curcumin in control vehicles and NEs (mean ± 90% CI, *n* = 4). The formulations are defined in Table 1 and Table 2.

**Control Vehicle**	***S*_V_ (mg/mL)**	***J*_SS_ (µg/cm^2^/h)**	***J*_max_ (µg/cm^2^/h)**	***S*_SC_ (mg/mL)**	***k_p_* (cm/h.1E5)**	D* (cm/h.1E5)	***k*_SC_**	
C1	0.7 ± 0.1	0.005 ± 0.001	0.005 ± 0.001	0.12 ± 0.01	0.80 ± 0.15	4.2 ± 0.8	0.18 ± 0.02	
C2	16.3 ± 1.3	0.020 ± 0.005	0.050 ± 0.016	3.10 ± 0.56	0.30 ± 0.01	1.6 ± 1.0	0.19 ± 0.03	
C3	3.7 ± 0.1	0.007 ± 0.001	0.007 ± 0.001	0.37 ± 0.06	0.19 ± 0.02	2.0 ± 1.0	0.10 ± 0.02	
C4	1.7 ± 0.1	0.015 ± 0.004	0.015 ± 0.004	0.26 ± 0.06	0.88 ± 0.24	5.7 ± 1.6	0.15 ± 0.04	
C5	0.8 ± 0.1	0.008 ± 0.002	0.008 ± 0.002	0.11 ± 0.01	0.92 ± 0.18	7.0 ± 1.4	0.13 ± 0.02	
Ce	0.8 ± 0.1	0.210 ± 0.020	0.210 ± 0.020	1.00 ± 0.20	26.0 ± 2.50	21.0 ± 2.0	1.24 ± 0.19	
**Nanoemulsion**	***S*_V_ (mg/mL) ^a^**	***J*_SS_ (µg/cm^2^/h)**	***J*_max_ (µg/cm^2^/h) ^a^**	***S*_SC_ (mg/mL) ^a^**	***k_p_* (cm/h.1E5)**	D* (cm/h.1E5)	***k*_SC_**	***ER***
N1	58.3 ± 5.90	0.33 ± 0.08	3.90 ± 1.00	4.30 ± 0.70	6.6 ± 1.9	89.0 ± 25.0	0.07 ± 0.01	18.6
N2	51.9 ± 2.0	0.12 ± 0.03	1.26 ± 0.36	6.00 ± 0.92	2.4 ± 0.7	21.0 ± 6.0	0.12 ± 0.02	6.0
N3	26.7 ± 0.3	0.49 ± 0.11	2.32 ± 0.52	8.70 ± 0.41	9.1 ± 2.0	30.0 ± 6.7	0.33 ± 0.02	11.0
N4	51.2 ± 4.7	0.46 ± 0.10	4.66 ± 1.00	6.20 ± 0.70	9.8 ± 2.2	75.0 ± 17.0	0.12 ± 0.01	22.2
N5	14.4 ± 0.8	0.23 ± 0.06	0.67 ± 0.18	6.13 ± 0.34	4.7 ± 1.3	11.0 ± 3.0	0.43 ± 0.02	3.2
N6	13.8 ± 0.2	0.19 ± 0.03	0.52 ± 0.07	5.95 ± 0.29	3.8 ± 0.5	9.0 ± 1.2	0.43 ± 0.02	2.5
N7	10.8 ± 0.1	2.66 ± 0.31	5.80 ± 0.67	3.60 ± 0.34	53.3 ± 6.3	161.0 ± 19.0	0.33 ± 0.03	27.6
N8	18.6 ± 0.2	1.19 ± 0.15	4.42 ± 0.57	4.30 ± 0.55	23.8 ± 3.1	103.0 ± 13.0	0.23 ± 0.03	21.0
N9	13.8 ± 0.1	0.26 ± 0.01	0.70 ± 0.01	7.39 ± 0.53	5.1 ± 0.1	9.5 ± 0.2	0.54 ± 0.04	3.3
N10	10.9 ± 0.7	0.37 ± 0.04	0.81 ± 0.09	6.83 ± 0.65	7.0 ± 0.8	11.8 ± 1.3	0.63 ± 0.06	3.9

^a^*p* < 0.0001 for N1-N10 compared to 60% ethanol (Ce). ER represents the enhancement ratio of *J_max_* for NE formulations compared to Ce.
